# Impact of Youth Community Health Volunteers on Community Health Screening Program Outcomes for Older Adults: Mixed Methods Evaluation Study

**DOI:** 10.2196/75699

**Published:** 2025-12-08

**Authors:** Ka Shing Yow, Audrey Shu Ting Kwan, Xiaoting Huang, Jie Xin Lim, Meng Han Lim, Lynn Pei Zhen Teo, Juliana Shariq Mujtaba, Muhammad Razzan Razaki, Yihan Khoo, Si Qi Lim, Alicia Shi Yao Chee, Jed Jasman, Jasmine Yee Ru Cheng, Elliott Weizhi Sim, Thaddeus Chi En Cheong, Nerice Heng Wen Ngiam, Angeline Jie-Yin Tey, Yu Heng Kwan, Chee Hsiang Liow, Lian Leng Low, Kennedy Yao Yi Ng

**Affiliations:** 1 Department of Internal Medicine, National University Health System, Singapore; 2 TriGen, Singapore; 3 Division of Population Health and Integrated Care, Singapore General Hospital, 10 Hospital Blvd168582, Singapore, 6562223322; 4 Department of Physiotherapy, Singapore General Hospital, Singapore; 5 Department of Geriatric Medicine, Singapore General Hospital, Singapore; 6 Primary and Community Care Divison, Ministry of Health, Singapore; 7 Department of Transitional Care and Community Medicine, Sengkang General Hospital, Singapore; 8 Department of Respiratory and Critical Care Medicine, Tan Tock Seng Hospital, Singapore; 9 Program in Health Services and Systems and Research, Duke-NUS Medical School, Singapore; 10 Centre of Population Health and Implementation Research, SingHealth Regional Health Services, Singapore; 11 Department of Rheumatology and Immunology, Singapore General Hospital, Singapore; 12 Saw Swee Hock School of Public Health, National University of Singapore and National University Health System, Singapore; 13 Research and Translational Innovation Office, SingHealth Community Hospitals, Singapore; 14 Division of Medical Oncology, National Cancer Centre Singapore, Singapore; 15 Department of Global Health and Population, Harvard T.H. Chan School of Public Health, Boston, MA, United States; 16 Oncology and Medicine Academic Clinical Program, Duke-NUS Medical School, Singapore

**Keywords:** youth community health volunteers, older adult, community health, preventive health, intergenerational program, digital coaching, digital literacy

## Abstract

**Background:**

Community health screening programs frequently report inconsistent follow-up rates and barriers to sustained lifestyle changes. HealthStart is a Self-Determination Theory (SDT)-based intervention that aims to increase the autonomy and competence of participants via volunteer engagement, health and digital coaching, and posthealth screening follow-up. Youth community health volunteers (YCHVs) were taught principles of motivational interviewing, health coaching, and the social determinants of health through a program model anchored in principles of intergenerational and service learning. YCHVs, guided by health care volunteers, served as health and digital advocates for participants over a 3-month postscreening period.

**Objective:**

This study aims to evaluate the effectiveness and acceptability of the HealthStart program, a structured, layperson-led intergenerational health coaching intervention.

**Methods:**

This study used a convergent parallel mixed methods design. A total of 192 older adult participants’ (mean age 66.9, SD 9.6 years) quantitative data were collected through pre- and postprogram surveys between September 2022 and January 2024. Follow-up with primary care provider (PCP) was the primary outcome; secondary outcomes include health goal attainment, self-efficacy, and digital and health literacy among older adults, and acceptability among all stakeholders. PCP follow-up and health goal attainment were self-reported, while self-efficacy was measured using the Patient Activation Measure-13. Health and digital literacy were assessed with an adapted questionnaire and the eHealth Literacy Scale (eHEALS), respectively. A total of 36 semistructured interviews were conducted with 13 older adults, 17 YCHVs, and 6 health care volunteers in the qualitative study between November 2023 and January 2024, which were qualitatively analyzed using thematic analysis. A joint analysis was conducted to generate meta-inferences.

**Results:**

The follow-up rate among PCPs increased significantly from 42.7% to 84.5% (*P*<.001). A total of 66.2% (92/139) of the participants achieved their health goals, and 81.3% (113/139) reported satisfaction with the program. There were no significant pre-post differences in Patient Activation Measure-13, knowledge, and eHEALS scores. However, a statistically significant correlation was found between postcycle eHEALS scores and the number of follow-up visits (*P*=.003). The qualitative findings substantiate HealthStart’s Theory of Change and its SDT underpinnings. Participants’ narratives highlighted the 3 SDT psychological needs, increased autonomy, competence, and relatedness, which are associated with sustained behavior change and health engagement. An increase in the PCP follow-up rate was influenced by individually tailored goal setting and relationship-building that leveraged motivational interviewing to support intrinsic motivation. The program’s primary outcome was, to some extent, influenced by digital health onboarding and possibly improvements in health literacy. Participants’ self-efficacy could have been enhanced with a structured social prescription framework.

**Conclusions:**

HealthStart demonstrated the feasibility of using YCHVs and SDT principles to improve PCP follow-up rates and promote healthier lifestyles through digital enablement among older adults participating in community health screening.

## Introduction

Many countries are facing an aging population [1], with a rising prevalence of chronic diseases [2]. Community health screenings are key preventive health programs designed to screen, detect, and manage these conditions. However, these screening programs report inconsistent follow-up rates [3,4], and many patients face barriers to sustaining lifestyle changes, including a lack of knowledge and inadequate community support [5,6]. In the United States, many cancer screening programs involve fewer than 75% of patients receiving some form of follow-up care [7]. In 2016, SingHealth, Singapore’s largest public health care cluster, reported that 1 in 4 individuals screened via community-based health screenings had not returned for a doctor’s follow-up after a year [8]. Loss to follow-up has significant physical and socioeconomic implications, including untreated chronic conditions, physical complications, reduced efficiency of the health care system, and increased financial costs [9].

These patterns reveal a critical gap in how traditional screening models are designed, focusing on biomedical detection but often overlooking the motivational, relational, and digital drivers of health behavior change. Research increasingly points to the importance of psychosocial support and person-centered coaching, and digital enablement in facilitating sustainable health outcomes [5,10]. Community health volunteers, when appropriately trained, have demonstrated potential to complement health systems by engaging individuals, reinforcing health messages, and bridging gaps in service delivery [11,12]. Community health volunteers can be empowered to guide health screening participants in navigating the health system, increasing follow-up rates, setting specific health-related lifestyle goals, and adhering to them. Previous qualitative studies have shown the benefits of this approach, including improved person-focused coordinated care [13], more seamless referrals to appropriate services [14], and the promotion of healthy practices [15]. Experts recommend measuring indicators that predict the success of lay counselors [16] and exploring their effectiveness in more complex tasks, such as diagnosis and counseling [17]. When designed and implemented appropriately, community-based health coaching has the potential to be a significant nonclinical intervention for individual and population health [18], simultaneously improving the health literacy of health coaches undergoing training and that of the general public, and facilitating the adoption of healthy practices across generations [19]. Despite promising findings, the literature on community health volunteers remains largely focused on adult volunteers, with limited research examining the effectiveness of youth community health volunteers (YCHVs), particularly those aged 15‐35 years, in the context of community-based health interventions or screening programs. While youth involvement in aging and intergenerational initiatives is well-documented, many of these programs are broad in focus and do not center specifically on health promotion [20-22]. The World Health Organization defines youth as individuals aged 15‐25 years, but in Singapore, the Ministry of Culture, Community and Youth adopts a broader definition, classifying youth as those aged 15-35 years [23]. For the purpose of this study, we adopt the latter definition.

In tandem, digital literacy has emerged as a key determinant of access to health information and services, especially among older adults [24,25]. This is particularly salient in health systems such as Singapore’s, where national preventive health reforms promote primary care engagement and follow-up via digital platforms [26,27]. However, many seniors face barriers to adopting digital tools, such as limited confidence, lack of guidance, or unfamiliarity with technology [28-30]. These barriers risk exacerbating digital exclusion, potentially widening health disparities [31,32]. As such, there is an urgent need for community-based digital health support integrated with preventive health efforts. YCHVs may be well-positioned to address this dual challenge by providing motivation-based coaching alongside digital enablement.

HealthStart was developed as a response to these gaps. Anchored in Self-Determination Theory (SDT), the program aims to address the core psychological needs of autonomy, competence, and relatedness in health behavior change. HealthStart operationalizes these principles through intergenerational service learning, where YCHVs serve as health coaches guiding older adults through a structured journey of goal-setting and lifestyle change by equipping them with digital health tools and skills.

In this study, we evaluated HealthStart as both a service delivery model and a motivation-based intervention. We used a mixed methods design with independently collected quantitative and qualitative data during the same timeframe, integrated post hoc through joint analysis to assess its impact on primary care providers’ (PCP) follow-up rates, health goal attainment, self-efficacy, and digital and health literacy among older adults. We also explored the perspectives of key stakeholders, including YCHVs, health care volunteers (HCV), and participants, to understand the program’s feasibility, acceptability, and areas for refinement.

## Methods

### Conceptual Framework

HealthStart is an SDT-based intervention designed to support the Healthier SG initiative, the local government’s policy to promote healthier aging by reshaping the population’s health-seeking behaviors and lifestyle through collaborative, community-based care [33]. The national reform also emphasizes digital inclusion, aiming to enroll seniors with a PCP through the national digital health apps. The program aims to increase the engagement and follow-up rates of participants attending a chronic disease community health screening by developing, training, and empowering YCHVs to serve as health and digital advocates who accompany participants on their postscreening journey over 3 months.

SDT proposes that personally relevant goals are more internally motivated and are thus more likely to be obtained than goals set due to some external pressure [34]. It specifies 3 basic psychological needs (ie, autonomy, competence, and relatedness) that provide the basis for motivation and development and are the contextual conditions that facilitate internal motivation and help people integrate their behavior into their everyday lives and sense of self. Autonomy refers to the feeling that one’s actions are the result of one’s own volition, competence is the belief in one’s ability to effect change and achieve desired outcomes, and relatedness is the extent to which one feels a connection with others. Self-integration of behaviors occurs when externally motivated behaviors (ie, behaviors regulated by an external force) become integrated into one’s sense of self; that is, they contribute to one’s overall self-evaluation.

Autonomy is fostered through health coaching and the cocreation of health-related lifestyle goals using the SMART framework (Specific, Measurable, Achievable, Realistic/Relevant, and Time-bound) [35]. Competency is developed through health education, action planning, and real-time feedback, alongside digital enablement (eg, using health apps to schedule appointments, access results, and track lifestyle goals). Relatedness is cultivated through continuity of follow-up with a dedicated YCHV. These shared decision-making mechanisms constitute the hypothesized mediators linking program activities to behavioral and clinical outcomes.

HealthStart is also anchored on 2 complementary frameworks (intergenerational and service-learning frameworks). YCHVs are taught health knowledge and health coaching skills, which they apply through their interaction with older adults under the guidance of HCVs. This program builds on prior intergenerational program experiences [36]. It facilitates intergenerational transfer of health knowledge between the different participants by incorporating principles of contact theory (institutional support, equal status, cooperative interaction, and shared goals) [37]. In addition, HealthStart facilitates the intergenerational transfer of health knowledge when YCHVs share the knowledge and experience gained from the program with their families and communities [38]. The program uses a service-learning approach, combining learning objectives with community service to create a meaningful learning experience that addresses the needs of older adults in the community [39,40]. Volunteers’ learning and development are facilitated through guided critical reflection, grounded in Kolb’s experiential learning cycle, which links their community service experience to learning objectives [39,40]. Finally, HealthStart leverages key components of a previously described digital literacy program shown to be effective in helping older adults acquire digital skills (eg, personalizing digital skills taught according to participants’ goals, having a tiered curriculum, using an aide-memoire, and connecting participants to a digital community) [41-43].

**Figure 1.**
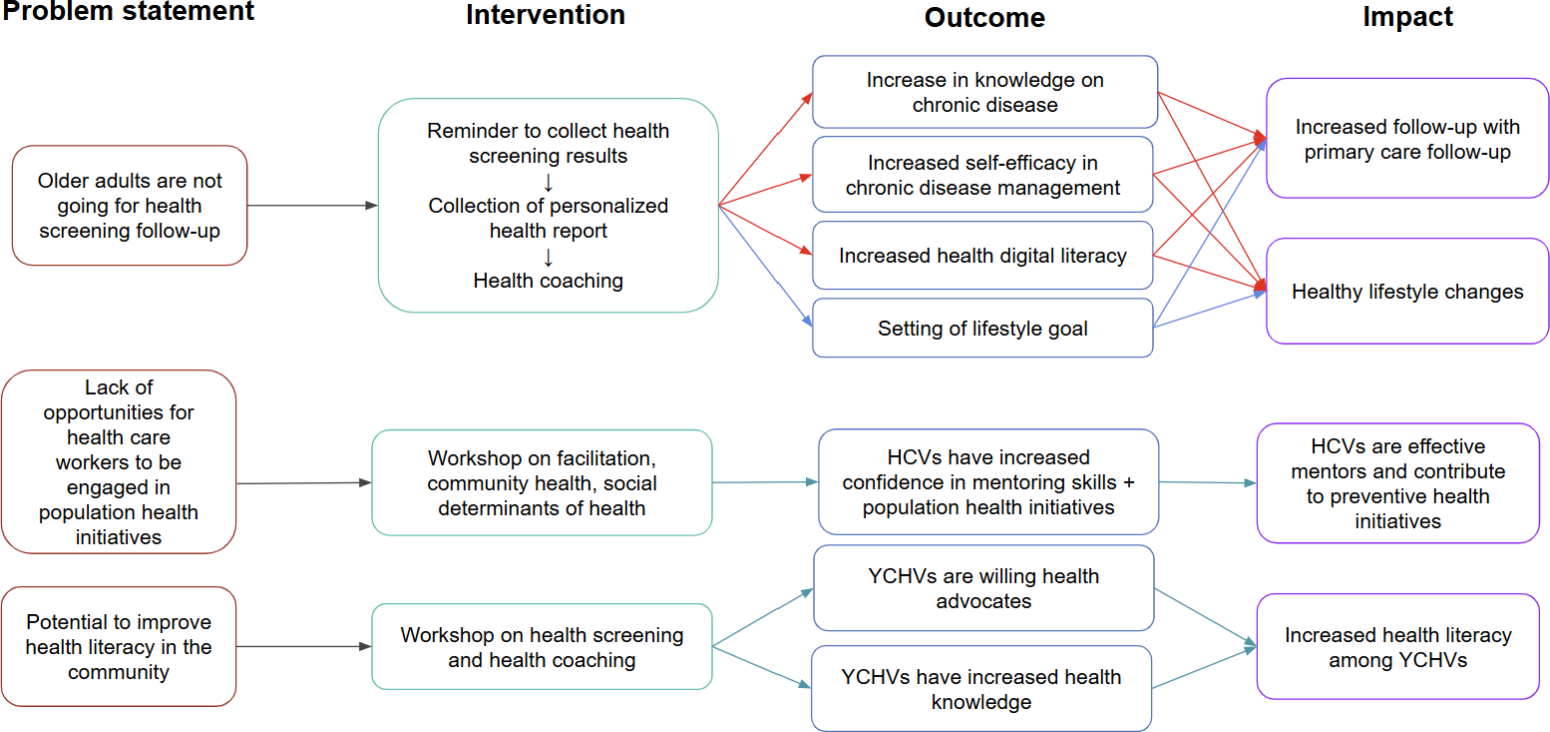
HealthStart theory of change. HCV: health care volunteer; SDT: Self-Determination Theory; SMART: Specific, Measurable, Achievable, Realistic/Relevant, and Time-bound; YCHV: youth community health volunteer. Red arrows denote Self-Determination Theory, blue arrows denote Specific, Measurable, Achievable, Realistic/Relevant, and Time-bound goals, and green arrows denote intergenerational and service learning.

The program’s theory of change is reflected in Figure 1.

### Program Implementation

For the participants, the program consisted of a personal review of their health screening results, health goal setting with their assigned YCHVs, follow-up with their PCPs, and completion of their health goals over a 3-month duration. Throughout the process, HCVs oversaw the operations and provided avenues for content clarification and professional support as necessary. Each HealthStart group consisted of 1 HCV and 4 YCHVs. Each YCHV was assigned 2 participants.

To complete the HealthStart program, participants would need to fulfill the following criteria:

1. Enrollment with a PCP.
2. Learn about their chronic diseases with the aid of the Health Promotion Board booklets [44].
3. Learn at least 1 digital health app, such as HealthHub, Healthy 365 [45,46].
4. Set a health-related lifestyle goal and achieve it.

The participants would first meet their YCHVs at a community event where health screening reports were distributed and explained by a community nurse. The YCHVs would conduct their first session of health coaching with the participants and build rapport via questions that covered sociodemographic factors and lifestyle aspects (exercise, smoking, alcohol intake, and dietary habits). YCHVs subsequently taught participants about their newly diagnosed chronic disease via Health Promotion Board educational materials [44], guided them in the use of digital health apps [45], assisted in setting up an appointment with a PCP through the use of the digital health app HealthHub, and supported the participants in setting a lifestyle goal. YCHVs followed up with their participants one-on-one longitudinally over a period of 3 months through in-person or virtual visits (ie, video calls) every fortnight, where they checked the participants’ progress, motivated them to achieve their goals, and ensured the fulfillment of the program’s 4 goals. YCHVs attended monthly meetings with HCVs to reflect on their intergenerational and service-learning experience. Further details of the program’s implementation are available in an earlier publication [46].

### Program Outcomes

The primary outcome of the program was residents’ enrollment with a PCP. The secondary outcomes include health goal attainment, self-efficacy, and digital and health literacy among older adults, as well as the acceptability of the program among stakeholders.

### Study Design and Rationale

This study used a convergent parallel mixed methods design, guided by the framework proposed by Edmonds and Kennedy [47], to evaluate the effectiveness and acceptability of the HealthStart program. Quantitative and qualitative program evaluation data were collected, analyzed, and interpreted simultaneously to develop comprehensive insights into the program’s mechanisms and outcomes.

Overall, 3 key considerations guided the selection of a mixed methods approach. First, while quantitative measures can capture concrete outcomes, such as follow-up rates and goal attainment, qualitative inquiry is essential to understand the relational and motivational mechanisms underlying these changes. Second, the implementation of a novel community health program required a rich contextual understanding, alongside outcome measures, to inform future scaling efforts. Third, the integration of multiple data types allowed us to examine how SDT operated in practice through both measurable outcomes and lived experiences. Both quantitative and qualitative strands were concurrently collected from the same cohort of participants and treated with equal priority in explaining the program’s outcomes.

To ensure quality and coherence across both strands, we applied the Good Reporting of A Mixed Methods Study (GRAMMS) checklist [48]. The checklist ensured adequate rigor in sampling, design, integration procedures, and comparison between data types. This quality appraisal validated the credibility of emergent meta-inferences and highlighted areas of strength, such as high fidelity in YCHV training and participant engagement.

### Participants

Older adults aged 40 years and above who participated in the health screening, YCHVs aged 15‐35 years, and HCVs who participated in the program were eligible for the study. Individuals with medical comorbidities that affected their ability to communicate (eg, severe dementia) were excluded from the study. Individuals who could not speak English, Mandarin, Malay, or Tamil were also excluded.

### Quantitative Data Collection and Analysis

Quantitative data collection took place between September 2022 and January 2024. All eligible older adults attending the health screening were invited to participate in the study. Data were collected at the start of the first health coaching session and at the end of the program through a digital survey administered by a trained volunteer or member of the research team via FormSG (Government Technology Agency of Singapore), a secure, encrypted, web-based government form with the following predesigned fields:

1. Sociodemographic variables: age, sex, race, marital status, highest education level, and residential type.
2. Primary outcome: follow-up with a primary care doctor, measured as “Yes” or “No.”
3. Secondary outcomes:
  - Knowledge questionnaire: a 12-item true or false questionnaire on chronic conditions that is an abbreviated version of the 15-minute chronic disease questionnaire [44], adapted to the local context using information from the Health Promotion Board brochures.
  - Patient Activation Measure (PAM-13): a 13-item questionnaire measuring individuals’ ability to self-manage chronic disease through self-reported knowledge, skills, and confidence validated in the local context [49].
  - eHealth Literacy Scale (eHEALS): an 8-item questionnaire assessing respondents’ perceived skills in using information technology for health [50].
  - SMART [35] health goal attainment: self-rated achievement of lifestyle goals, measured as a “Yes” or “No.”
  - Program satisfaction: “To what extent are you satisfied with the program?” with 4-level Likert scale response options (not at all satisfied, slightly unsatisfied, slightly satisfied, and very satisfied).

Descriptive statistics were computed for the study population. The program implementation fidelity was tracked by logging the number of volunteer-participant encounters and the electronic survey responses (including engagement mode, duration, and coaching elements delivered) along with the number of final assessments conducted. The McNemar test was used to examine differences in the proportion of primary care follow-up before and after the program. Pre- and postintervention differences in secondary outcomes were compared using paired *t* tests. One-way ANOVA and linear trend tests were used in the subgroup analysis to evaluate the relationships between the secondary outcomes and the number of follow-up visits the residents received. A binomial logistic regression was performed with primary care follow-up as the outcome variable and demographics and baseline questionnaire scores as the independent covariates. All the statistical analyses were performed via Stata (version 15, release 15; StataCorp, 2017). Statistical significance was set at *P*<.05.

### Qualitative Data Collection and Analysis

Qualitative data were collected post-program through one-to-one, semistructured interviews with older adults, HCVs, and YCHVs. Purposive sampling was used to recruit older adults who completed the program and quantitative study, based on the following criteria to ensure the representativeness of each group: age, sex, race, education level, number of follow-up visits within the program, and postprogram primary care follow-up status. The entire cohort of HCVs and YCHVs was sampled; all HCVs and YCHVs who completed the program were approached and recruited for the study. The interview guides were formulated based on the theory of change to elicit perceptions of the program, the health advocacy process with older adults, and the volunteer training program (MultimediaAppendices 1^3^).

The interviews were conducted between November 2023 and January 2024, either face-to-face or via Zoom Web Conferencing (Zoom Video Communications, Inc.), by 4 project team members who were trained and supervised by the qualitative lead coinvestigator. Each interviewer was trained in qualitative data collection techniques. All interviews were audio-recorded and transcribed using an automated transcription software service, and then reviewed by a project member to ensure verbatim accuracy and redact identifiable information. The 4 team members coded the transcripts using a deductive-abductive approach, focusing on perceptions of the program, the health advocacy process, and volunteer training, while allowing new themes to emerge. Thematic analysis, following the framework of Braun and Clarke [51], was used by 4 coders to analyze the transcripts. After independently coding the transcripts, a secondary coder reviewed them to address discrepancies and finalize the coding. QSR NVivo 14 software (Lumivero) was used for data management and analysis. Data triangulation was used to identify areas where qualitative themes aligned with quantitative data findings on program implementation fidelity and program outcomes.

The 4 team members affiliated with the research and evaluation arm of the Division of Population Health and Integrated Care at Singapore General Hospital were involved in the qualitative study. All of them are public health researchers or researchers-in-training with an interest in community health, and they acknowledged that their institutional roles and prior involvement in the program may have influenced the interpretation of the findings. To enhance reflexivity, interviewers engaged in pre- and postinterview debriefs, used semistructured guides to maintain consistency, and had responses cross-checked by multiple investigators to surface diverse perspectives. The team members remained attentive to potential power dynamics, reassured participants that their views would be received openly, and critically interrogated their positionality throughout data collection, analysis, and reporting to mitigate bias.

### Data Integration

**Figure 2.**
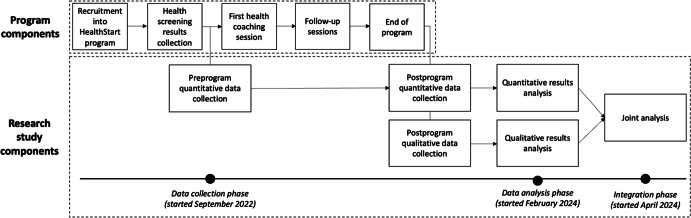
Flowchart of data collection and analyses. Program components span over a duration of 3 months. There were 2 iterations of the program in 2022 and 2023, respectively. Quantitative data were collected from participants pre and post program. Qualitative data were collected from participants, health care volunteers, and youth community health volunteers during the same phase as the postprogram quantitative data collection. The data were collected over the 2 iterations of the program.

The data analysis and integration process occurred in 3 phases (Figure 2). Quantitative and qualitative data were collected from the same cohort of participants in the same phase of the study. Regular meetings between project team members established preliminary findings from each strand. Thereafter, in the integration phase, joint displays were developed to map qualitative themes to quantitative outcomes, and patterns of convergence and divergence were systematically examined. Meta-inferences were generated with particular attention to how qualitative findings explained variations in quantitative outcomes. Validity was enhanced via iterative checks on integrated interpretations.

Quality assurance measures included the application of the Good Reporting of A Mixed Methods Study (GRAMMS) [48], regular team meetings to discuss emerging patterns, an independent review of integration conclusions, and systematic documentation of integration decisions. This rigorous approach to integration enabled us to develop a more nuanced understanding of program mechanisms than would have been possible through either method alone.

### Ethical Considerations

This project received approval from the SingHealth Centralised Institutional Review Board (CIRB reference number 2022/2700). All participants provided informed consent and were given a participant information sheet. Parental consent was obtained for participants below the age of 21 years. Individuals who took part in the qualitative interviews received a reimbursement of US $23. All quantitative and qualitative data collected were deidentified and stored on a set of electronic devices that were only accessible by the evaluation team. No data related to any individual, in any form, are included in this publication.

## Results

### Overview

**Figure 3.**
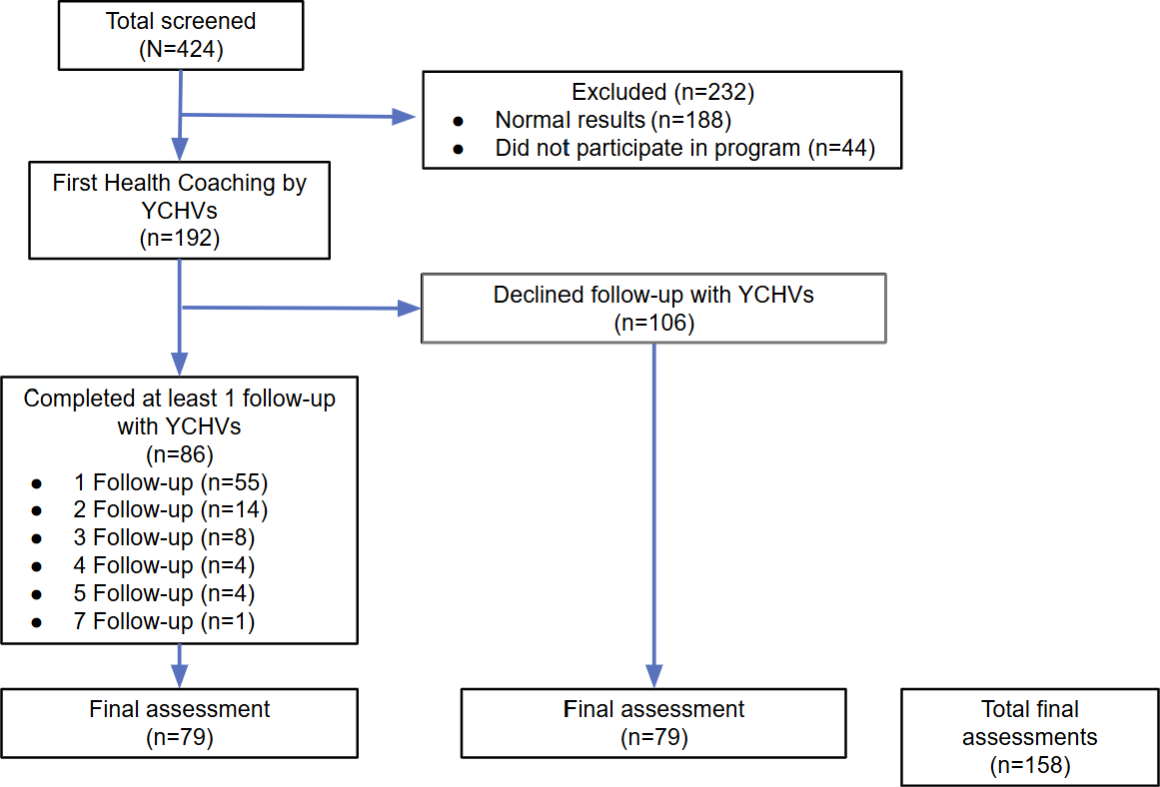
Flowchart of program participants.

HealthStart reached out to 192 older adults via 33 HCVs and 102 YCHVs over 2 health screening cycles conducted in 2022 and 2023, respectively. A total of 424 participants underwent the community health screening program, with 192 of them being eligible and subsequently recruited for the study. Figure 3 illustrates the flowchart of the program participants. Among the 192 study participants, 158 completed the study. The demographics of the study participants are summarized in Table 1. The mean age of the study participants was 66.9 (SD 9.6) years, and the sex distribution was approximately equal. Most of the participants were of Chinese ethnicity, had completed primary or secondary school, and resided in self-owned flats. A total of 36 semistructured interviews were conducted with older adults, HCVs, and YCHVs, achieving thematic saturation. The characteristics of the subjects can be found in Table 2.

**Table 1.** Demographics of the study participants.

Demographics	HealthStart participants (N=192)
Age (years), mean (SD)	66.9 (9.6)
Sex, n (%)	
Male	92 (48)
Female	100 (52.1)
Ethnicity, n (%)	
Chinese	170 (88.5)
Others	22 (11.5)
Marital status, n (%)	
Single	58 (30.3)
Married	133 (70)
Highest education level, n (%)	
No formal education	18 (9.38)
Primary education	52 (27.1)
Secondary education	79 (41.2)
Tertiary education	43 (22.4)
Status of residential or living status, n (%)	
Rent or lodge	27 (14.1)
Own	165 (85.9)
Resident questionnaires, mean (SD)	
Knowledge	8.81 (1.58)
PAM-13^a^	68.7 (17.9)
eHEALs^b^	24.5 (10.6)

a PAM-13: Patient Activation Measure-13.

b eHEALS: eHealth Literacy Scale.

**Table 2.** Demographics of the interviewees.

Demographics	Values
Older adults’ characteristics (n=13)	
Sex, n (%)	
Male	8 (61.5)
Female	5 (38.5)
Age (years), n (%)	
51‐60	2 (15.4)
61‐70	8 (61.5)
71‐80	1 (7.7)
>80	2 (15.4)
Ethnicity, n (%)	
Chinese	11 (84.6)
Malay	1 (7.69)
Indian	1 (7.69)
Marital status, n (%)	
Single, divorced, or widowed	6 (46.2)
Partner or married	7 (53.8)
Education, n (%)	
No formal education	1 (7.69)
Primary	5 (38.5)
Secondary	3 (23.1)
Tertiary	4 (30.8)
Residential status, n (%)	
Rent or Lodge	6 (46.2)
Own	7 (53.8)
Number of touchpoints with the program, n (%)	
1	3 (23.1)
2	2 (15.4)
3	3 (23.1)
4	1 (7.69)
5	3 (23.1)
6	1 (7.69)
Follow-up status with primary care doctor, n (%)	
Following up at baseline	5 (38.5)
Started following up after the program	5 (38.5)
Not following up after the program	3 (23.1)
YCHV^a^ characteristics (n=17)	
Age (years), n (%)	
<21	12 (70.6)
21‐35	5 (29.4)
Sex, n (%)	
Male	3 (17.6)
Female	14 (82.4)
HCV^b^ characteristics (n=6)	
Age (years), n (%)	
20‐30	1 (16.7)
31‐40	4 (66.7)
51‐60	1 (16.7)
Sex, n (%)	
Male	2 (33.3)
Female	4 (66.7)

a YCHV: youth community health volunteer.

b HCV: health care volunteer.

### Integrated Program Outcomes

Our mixed methods analysis revealed substantial program impacts across multiple domains, with strong convergence between quantitative outcomes and qualitative insights. The integration of findings provided a deeper understanding of the program’s mechanisms and contextual factors influencing its effectiveness. We present these findings through an integrated analysis of each outcome, highlighting areas of convergence and divergence between data types.

### PCP Follow-Up

Quantitative analysis demonstrated a significant improvement in PCP follow-up, increasing from 42.7% to 84.5% (*χ*²_1_=43; *P*<.001). This effect was particularly pronounced among older adults (OR 1.8, 95% CI 1.2‐2.7) and participants from minority ethnic groups (OR 1.6, 95% CI 1.1‐2.3).

Qualitative findings supported these findings. Older adults credited YCHVs with demystifying health screening results, reinforcing the importance of follow-up with a primary care doctor, and providing support that bolstered their commitment to seeing a doctor. This had a specific impact on often underserved groups of older individuals and minority ethnic groups, many of whom were not connected to primary care to begin with. One participant shared:

(YCHV) asked me to see [a] doctor… Then I go to poly(clinic), then I go check and take the medicine, cholesterol medicine, I take. Before that I never take. Before that (health screening) I never thought to see [a] doctor…

[OA13, Female, 60-70 years]

The integration of quantitative and qualitative data suggested that the relationship-based support model was instrumental in overcoming traditional barriers to health care access. YCHVs served as cultural bridges, combining practical assistance, such as appointment scheduling and reminders with emotional support. In doing so, they enhanced participants’ sense of competence in managing health information and navigating the system, while fostering autonomy by encouraging informed and self-directed health care choices. This finding was particularly notable among participants who had previously reported low engagement with the health care system.

### Health Behavior Change and Goal Attainment

A dose-response relationship was observed between the number of YCHV follow-up sessions and SMART goal attainment. A statistically significant linear-by-linear trend (Z=2.44; *P*=.02) was observed between health goal achievement and those with none (58.46%), 1 (65.91%), or 2 or more (87.5%) YCHV follow-up visits. A total of 66.2% (92/139) of participants achieved their health goals, and 81.3% (113/139) reported satisfaction with the program. There were no statistically significant changes in the scores before and after the intervention for knowledge (*t*_190_=0.418; *P*=.68) and PAM-13 (*t*_190_=−1.99; *P*=.05). There were no statistically significant changes in knowledge (*F*_2,130_=0.61; *P*=.54) or PAM-13 scores (*F*_2,130_=2.07; *P*=.13) via one-way ANOVA between groups stratified by the number of YCHV follow-up visits received.

Qualitative findings supported and deepened the understanding of these results. The participants mentioned achieving various lifestyle changes, including reducing the intake of unhealthy foods, increasing the consumption of healthier dietary options, and engaging in more physical activity. They reported additional health benefits that were not captured in the quantitative outcomes, such as weight loss, improvements in health markers, and an overall sense of well-being. While some older adults were unable to articulate specific facts on chronic diseases, this did not hinder their understanding of the importance of adopting healthier lifestyle choices. These behavior changes were often framed as internally motivated, goal-oriented efforts, consistent with HealthStart’s SDT framework. One participant described:

Eating vadai (savory Indian fried snack) … my favorite…I cut down a lot. One day Roti Prata (fried Indian flatbread) I also cut down eh… I think I changed a lot… What they told me I just listen, just keep in mind what they told me, just try my best… I feel that consulting with [YCHV], I feel [I had learnt] something

[OA02, Male, 60-70 years]

While many participants credited YCHVs for facilitating conversations that increased their awareness of healthy habits and motivation to make meaningful changes in their daily routines, specific measures or support systems to improve confidence and efficacy in managing one’s health were lacking for some participants. One participant described:

…about setting targets, there is no point… It is like I am in school and the teacher asked you to follow the timetable, would you?.,. If the patient doesn’t follow, it is of no use as well

[OA10, Male, 60-70 years]

Meta-inference development identified 3 key mechanisms of behavior change: (1) relationship-based accountability, (2) culturally contextualized health messaging, and (3) practical problem-solving support. These mechanisms were consistently present in successful cases, with more that can be done in the area of practical measures and support systems, as evidenced by both quantitative goal achievement data and qualitative narratives.

### Digital Health Literacy and Technology Adoption

Analysis of eHealth Literacy Scale (eHEALS) scores revealed a threshold effect in smartphone health app adoption via follow-up visits by YCHVs. While there was a statistically significant increase in eHEALS scores in the groups that experienced more follow-up sessions, as determined by one-way ANOVA (*F*_2,130_=6.06; *P*=.003), the Tukey post hoc test revealed a statistically significant increase in eHEALS scores in those with more than 1 YCHV follow-up (mean 30.4, SD 8.4; *P*=.002) compared with those with no follow-up (mean 23.5, SD 8.4) and no significant difference between those with more than 1 YCHV follow-up and those with only 1 YCHV follow-up (mean 26.3, SD 8.7; *P*=.15). Qualitative data deepened understanding in this area. While there were many participants who already had health apps on their phones and felt there was no significant impact from the program, a number were introduced and oriented to apps of practical use for scheduling appointments with PCPs, tracking, and accomplishing health goals, which they found beneficial.

The integration of findings suggested that digital health adoption followed a threshold effect, with meaningful improvements occurring only after onboarding, establishing rapport, and providing hands-on guidance.

### Stakeholder Experiences and Program Implementation

Overall, 81.3% (113/139) of the residents reported feeling “Satisfied” or “Very Satisfied” with the program. A summary of the above outcomes can be found in Table 2.

Qualitatively, older adults appreciated the program for several reasons, including the health knowledge and benefits gained, the support from YCHVs in achieving their health goals, the meaningful connections formed, the opportunity to participate free of charge, and the promotion of healthier lifestyles for both younger and older Singaporeans. The majority of YCHVs expressed that being a lay health volunteer was fulfilling and enriching. Many valued the meaningful connections formed with older adults, the ability to impact their health positively, and the opportunity to acquire health knowledge and interpersonal skills. Most YCHVs reported that they were keen to volunteer again in the program and would encourage their peers to participate. HCVs are also encouraged by YCHVs’ ability to impact older adults’ health positively and to have the opportunity to mentor them as effective health advocates.

Integration with quantitative satisfaction data revealed that program acceptability was linked to the quality of YCHV-participant relationships and the practical value of health coaching support. The joint analysis of both data types suggested that program success depended on both technical competence and interpersonal effectiveness of YCHVs.

Table 3 presents a joint display integrating quantitative results and qualitative themes. Meta-inferences generated through convergence and divergence analysis highlight the mechanisms of change observed in the HealthStart program, particularly the motivational impact of YCHVs’ coaching in reinforcing autonomy, competence, and relatedness.

Participants who dropped out of the program were analyzed to identify differences between those who completed the program and those who did not. Participants who dropped out of the program were statistically younger in age and had higher baseline PAM-13 scores (Table S5 in Multimedia Appendix 4). Individuals with higher baseline confidence in self-managing their health may not perceive the immediate benefits of the program and may therefore experience a higher dropout rate.

**Table 3.** Joint display of quantitative, qualitative, and meta-inference findings.

Outcome	Quantitative findings	Qualitative insights	Meta-inference (integrated interpretation)
Primary care follow-up	Increased from 42.7% preprogram to 84.5% post program (χ²_1_=43; *P*<.001). Older adults and minority ethnic groups are more likely to follow up on their care.	YCHVs^a^ facilitated follow-up by explaining results in lay terms, using participants’ mother tongues, and booking appointments. Older adults and minority groups felt reassured and more confident seeing their GP^e^.	Convergence: YCHVs’ personalized, culturally sensitive support improved primary care follow-up, especially among vulnerable groups. By clarifying results and assisting with appointments, they enhanced competence; by fostering trust and encouraging self-directed decisions, they supported autonomy, enabling greater health care engagement.
Health goal attainment	66.2% (92/139) achieved health goals; higher attainment with ≥2 YCHV follow-ups (*z*=2.44; *P*=.02)	Participants adopted healthier diets and physical activity routines. YCHVs used motivational interviewing and goal-setting frameworks to encourage behavior change.	Convergence: individualized goal-setting built participants’ autonomy and relatedness, translating into sustained health-related lifestyle changes.
Health knowledge	No significant pre–post differences in knowledge (*P*=.68) or when stratified by number of YCHV follow-up visits (*F*_2,130_=0.61; *P*=.54)	While older adults were unable to articulate specific facts on chronic diseases, this did not hinder their understanding of the importance of adopting healthier lifestyle choices.	Divergence: health awareness and practices improved despite unchanged knowledge scores, suggesting that formal knowledge tests may not fully capture learning achieved through relational engagement and experiential understanding. High baseline knowledge scores may also reflect a ceiling effect.
PAM-13^b^	Borderline significant pre-post difference in PAM-13 (*P*=.05) when stratified by number of YCHV follow-up visits (*F*_2,130_=2.07; *P*=.13)	Specific measures or support systems to improve confidence and efficacy in managing one’s health were lacking for some participants.	Convergence: the program lacks specific measures targeted at increasing self-efficacy. Social prescription could be a structured approach to address this inadequacy.
Digital health literacy (eHEALS)^c^	No significant change overall; improved among those with ≥1 YCHV follow-up (*F*_2,130_=6.06; *P*=.003)	Mixed responses; most older adults had familiarity with health apps, but some required onboarding. Digital health adoption required personalized support.	Convergence: digital health adoption followed a threshold effect, with meaningful improvements occurring only after onboarding, establishing rapport, and providing hands-on guidance.
Acceptability and program satisfaction	81.3% (113/139) reported being satisfied or very satisfied.	Older adults and YCHVs valued HealthStart as personable, supportive, and culturally accessible. YCHVs felt well-prepared and found the experience fulfilling. HCVs^d^ noted that the program enhanced patient-centered care and strengthened follow-up.	Convergence: positive quantitative ratings and rich qualitative testimonials underscore HealthStart’s acceptability and relevance to stakeholders.

a YHCV: youth community health volunteer.

b PAM-13: Patient Activation Measure-13.

c eHEALS: eHealth Literacy Scale.

d HCV: health care volunteer.

e GP: general practitioner.

## Discussion

### Principal Findings

This mixed methods evaluation of HealthStart demonstrated that a structured, layperson-led, intergenerational health coaching intervention program was both feasible and acceptable to older adults and YCHVs, and effective at improving primary care follow-up rates and supporting behavior change. Our analysis revealed both the measurable impacts of the intervention and the underlying mechanisms that facilitated these changes, while also highlighting important considerations for future community health initiatives.

### Primary Outcome

The postprogram PCP follow-up rate was 84.5%, higher than previously reported rates in health screening programs [6,7], with a significant increase among older participants and minority ethnicities. Older adults and minority race groups have been shown to have lower screening follow-up rates than younger age and majority race groups in previous studies [52]. HealthStart deliberately linked YCHVs with older adults who spoke the same mother tongue and provided an educational booklet in the older adult’s mother tongue to facilitate accessibility, communication, and understanding, which likely helped reduce age and racial inequity. This finding is consistent with those from similar community health initiatives [53], which underscore the importance of culturally and linguistically appropriate support.

### Integrating Study Findings With the Theory of Change

By mapping quantitative outcomes onto the Theory of Change and examining qualitative data for mechanisms of action, we offer insight into why HealthStart achieved its desired outcomes.

Setting a lifestyle goal was a mandatory requirement for all participants. The health goal attainment rate was 66.2%, with individuals being more likely to achieve them if they had more YCHV follow-ups; the majority of goals centered on improving one’s diet or physical activity. A total of 54.5% of a group of 33 participants achieved their set health goal in a previous community outreach health coaching program conducted by certified health coaches in Missouri [54]. This study revealed similar rates of health goal attainment among older adults who received abnormal health screening results, with their postscreening journey facilitated by YCHVs. The qualitative findings highlighted a key takeaway of the program being understanding, planning for, and achieving healthy lifestyle changes, facilitated by health coaching. As encompassed by the SDT framework [34], the program built on residents’ autonomy through individualized goal-setting and competence through health knowledge, self-efficacy, and digital health literacy to achieve health-related lifestyle changes.

Sharing knowledge about their health condition, tips on how to manage it, and onboarding relevant health digital apps was a core component of the curriculum YCHVs underwent and subsequently engaged participants with during their service in HealthStart. While there was no significant change in the pre-post scores in knowledge, qualitative analysis revealed that participants shared increased awareness and practice of healthy habits in domains such as diet and exercise. The participants in this study were noted to have high baseline knowledge (8.81/12, SD 1.58) and high baseline PAM-13 scores (68.8/100, SD 1.56), reflecting readiness to adopt new behaviors but potentially having difficulty sustaining change. The lack of a statistically significant difference could also reflect a ceiling effect of both scores [55]. Additionally, research shows that only specific aspects of higher level health literacy, such as communicative and critical health literacy, are associated with health-related lifestyle behaviors [56]. A knowledge score on general chronic conditions may not have reflected this change that occurred in participants. The qualitative analysis also revealed areas in which the program could be improved in supporting participants’ confidence and self-efficacy. A key recommendation was to consider social prescription, a structured system that allows YCHVs to link participants to community resources that can better support and sustain their goals for healthy living [57], which is planned to be implemented in future iterations of the program.

While there was no significant overall improvement in digital health literacy scores, subgroup analysis showed improvement among those with ≥1 YCHV follow-up (*F*_2,130_=6.06; *P*=.003). The findings suggest that onboarding digital health apps may be sufficient to impact health behavior. The qualitative findings support this; several participants set goals to learn digital health apps so that they could track their health-promoting behaviors or connect with a group or health system to sustain healthy behaviors. Previous research corroborates the need for initial support and familiarization with digital apps for older adults, as this lowers the barrier for adoption of digital health, which in turn can reshape health-seeking behaviors and lifestyles [26,30]. Therefore, the integration of digital health coaching into HealthStart reflects not only a programmatic innovation but also a response to national and global priorities around digital inclusion for older adults. As platforms like HealthHub become increasingly central to Singapore’s preventive health ecosystem, programs like HealthStart offer a scalable and person-centered model to bridge the digital divide and empower seniors to take greater ownership of their health.

Our findings substantiate HealthStart’s Theory of Change and its SDT underpinnings. Participants’ narratives highlighted the 3 SDT psychological needs, increased autonomy, competence, and relatedness, which are associated with sustained behavior change and health engagement. The increase in PCP follow-up rate was primarily influenced by individually tailored goal setting and relationship building, which leveraged motivational interviewing to support intrinsic motivation. The program’s primary outcome was, to a lesser extent, influenced by digital health onboarding and possibly improvements in health awareness and practices. Participants’ self-efficacy could have been enhanced with a structured social prescription framework.

### Acceptability and Feasibility

High satisfaction scores were reported by older adults participating in the program (81.3%), and qualitative reports demonstrated the acceptability and appreciation for the program from both volunteers and older adults. YCHVs felt it was a rich experience and expressed interest in volunteering in similar programs again. Older adults valued the new health insights gained and reported improvements in their health and lifestyles. A qualitative study in Canada, which integrated community volunteers as extensions of the primary care team to support older adults, yielded similar findings. These community volunteers deepened the primary care team’s understanding of their patients and facilitated the delivery of person-centered care, demonstrating the potential for integrating community volunteers into the primary care setting [13]. A separate study conducted in Canada revealed that community volunteers can play a role in increasing older adults’ understanding of community resources and navigating the system [14]. YCHVs are potentially untapped resources that can effectively engage and promote healthier living among older adults.

Given the effectiveness, acceptability, potential reach, and adoption of HealthStart, the evaluation demonstrates the potential to scale community programs involving laypersons in population health initiatives [58]. Program sustainability is further strengthened by collaborative work with local grassroots organizations and alignment with the national strategic context of promoting healthier aging [59]. However, our findings also highlight the importance of adequate volunteer training, attention to cultural and linguistic matching, and the potential application of a structured social prescription framework based on individualized preintervention health and social needs to promote sustainable health behaviors.

### Strengths and Limitations

This study’s mixed methods approach is a notable strength, allowing triangulation of data to capture both the breadth and depth of HealthStart’s impact, including experiences that standard instruments cannot easily quantify. However, this study has several limitations. First, the observed improvements could not be attributed to the program due to the absence of a control group. More rigorous designs, such as quasi-experimental or step-wedge trials, can be considered in future research to establish causality. Second, the majority of the studies’ quantitative outcomes were based on self-reported scales. This may not have adequately captured the depth and nuance behind participants’ motivation and progress as described in the qualitative arm. Future studies may adopt a sequential design with follow-up qualitative research conducted for each quantitative finding and corroborate the findings with other objective data, such as electronic medical records, biometric measures (eg, blood pressure, weight, and HbA_1c_), field observations, and multimodal assessments, including neurophysiological changes, cognitive, behavioral outcomes, and integration of caregiver perspectives as secondary informants that enhance understanding of health impact to older adults beyond traditional measures [60]. Additionally, selection bias may be present in YCHVs who completed the program, potentially influencing overall program outcomes such as goal achievement, participant, and volunteer satisfaction.

### Conclusions

This study demonstrated the feasibility of using YCHVs and SDT principles to improve PCP follow-up rates and promote healthier lifestyles among older adults participating in community health screening. The high acceptability of this model among stakeholders highlights the potential of harnessing laypersons, an untapped and valuable community resource, in population health initiatives.

## Supplementary material

10.2196/75699 Multimedia Appendix 1 HealthStart older adult topic guide.

10.2196/75699 Multimedia Appendix 2 HealthStart health care volunteer topic guide.

10.2196/75699 Multimedia Appendix 3 HealthStart youth community health volunteer topic guide.

10.2196/75699 Multimedia Appendix 4 Subgroup analysis of participants based on completion of the HealthStart Program.

10.2196/75699 Checklist 1 Good Reporting of A Mixed Methods Study (GRAMMS) Checklist.

## Abbreviations

eHEALS: eHealth Literacy Scale
GRAMMS: Good Reporting of A Mixed Methods Study
HCV: health care volunteer
HPB: Health Promotion Board
PAM-13: Patient Activation Measure-13
PCP: primary care provider
SDT: Self-Determination Theory
SMART: Specific, Measurable, Achievable, Realistic/Relevant, and Time-bound
YCHV: youth community health volunteer
