# Supplementary material for: Impact of Youth Community Health Volunteers on Community Health Screening Program Outcomes for Older Adults: Mixed Methods Evaluation Study
Source: J Med Internet Res. 2025 Dec 8;27:e75699. doi: 10.2196/75699 (PMC12685235; doi:10.2196/75699)
Supplement: Multimedia Appendix 1 [file jmir-v27-e75699-s001.docx]

**Table S1.** HealthStart older adult topic guide

| **Participant Background** | 1. Which estate do you live in? 2. When did you attend the screening program organized by Singapore General Hospital? |
| --- | --- |
| **Perceptions of Program & Outcomes** | 1. What was your experience going through the program? |
|  | 1. How did you follow up with a GP after the program?    1. If yes, what led you to follow up?    2. If no, what were the barriers?    3. How was this decision influenced more by volunteers? |
|  | 1. Can you share about the healthy lifestyle goals you set? (e.g., diet, exercise)    1. Were you able to achieve/maintain them?    2. Why or why not?    3. How were these influenced by volunteers or the screening? |
|  | 1. How did you understand your screening results?    1. How serious did you think they were?    2. Were any results new to you?    3. Which aspects of the program helped? |
|  | 1. Have you used digital health apps (e.g., HealthHub, Healthy365, Singpass), and did this change after the program?    1. If yes, what supported or hindered your use?    2. Were changes influenced by volunteers or the screening? |
|  | 1. How confident are you to take steps to care for your health?    1. Has your confidence changed after the program?    2. Was this influenced by volunteers or the screening?    3. How confident are you to continue these behaviors without follow-up? |
| **Perceptions of YCHVs** | 1. How was your experience interacting with the YCHVs?    1. How effective/helpful were they?    2. What was your favorite and least favorite part?    3. How could the experience be improved?    4. What are your thoughts on having youth volunteers in this role? |
| **Program Recommendations** | 1. What did you like most about the program? 2. How could it be improved?    1. Suggestions to support doctor follow-up?    2. Suggestions to support healthy lifestyle choices? |
| **Closing** | 1. Is there anything else you would like to share that we have not covered? |
