# Supplementary material for: Impact of Youth Community Health Volunteers on Community Health Screening Program Outcomes for Older Adults: Mixed Methods Evaluation Study
Source: J Med Internet Res. 2025 Dec 8;27:e75699. doi: 10.2196/75699 (PMC12685235; doi:10.2196/75699)
Supplement: Multimedia Appendix 2 [file jmir-v27-e75699-s002.docx]

**Table S2.** HealthStart health care volunteer topic guide

| **Participant Background** | 1. Can you share about what you do for your day job? 2. How was your experience volunteering with HealthStart? 3. Can you share about what you did as a healthcare volunteer?    1. Who do you work with?    2. *(Prompts:* Residents within a specific locality, specific conditions, n*on-healthcare volunteers*) |
| --- | --- |
| **Volunteering Processes and Experiences** | 1. All the healthcare volunteers received training at the start of the program. What were your thoughts about the training?    1. *(Prompts*: mentoring, facilitation skills, training for motivational interviewing, health coaching)    2. What did you gain most out of it?    3. How did it influence your mentoring of the non-healthcare volunteers?    4. How did it influence your perception of population health initiatives?    5. What was the least helpful?    6. What can be improved? 2. Why did you sign up as a volunteer?    1. Are those goals met?    2. What do you enjoy most about being a volunteer?    3. What do you enjoy least about being a volunteer?    4. How long do you see yourself continuing to volunteer in such a programme?    5. In what way can the volunteering programme be improved to better support you in your volunteering role? |
| **Program Objectives and Recommendations** | 1. What did you like most about this program? 2. How do you think the programme can be improved? Do you have any suggestions on what can be done to help people follow up with their doctor and make healthier lifestyle choices?    1. Is there anything you feel that you would need that will help you in your volunteering role? |
| **Additional Information** | 1. Is there anything else that we have not covered that you would like to share? |
