# Supplementary material for: Impact of Youth Community Health Volunteers on Community Health Screening Program Outcomes for Older Adults: Mixed Methods Evaluation Study
Source: J Med Internet Res. 2025 Dec 8;27:e75699. doi: 10.2196/75699 (PMC12685235; doi:10.2196/75699)
Supplement: Multimedia Appendix 3 [file jmir-v27-e75699-s003.docx]

**Table S3.** HealthStart youth community health volunteer topic guide

| **Participant Background** | 1. Can you share what you normally do for your day job/your studies? |
| --- | --- |
| **Volunteering Processes and Experiences** | 1. What was your experience volunteering with HealthStart?    1. What was the follow-up experience with your resident?    2. HealthStart aims to link residents with abnormal health screening results to a primary care provider. Could you describe how you helped with the follow-up with a doctor? What were some challenges faced?    3. Could you describe how you helped increase knowledge and awareness on chronic disease awareness on chronic disease for your resident? How do you facilitate this process?    4. One of the aims of the programme is to increase self-efficacy in the health domain for the residents. How did you assist your resident in this area?    5. Could you share if your resident managed to pick up digital skills to enhance health monitoring or management (e.g. onboarding HealthHub)? How did he/she do so? What were some challenges faced, and how did you overcome them?    6. Could you describe the process in which your resident planned his/her lifestyle goal? How did you facilitate the process? Any challenges? 2. All the volunteers received training at the start and were taught to conduct motivational interviewing and health coaching. What were your thoughts about the training?    1. How did the training impact your health knowledge?    2. How did this training change how you approached the resident for health coaching?    3. What was the most helpful?    4. What was the least helpful?    5. What can be improved? 3. To what extent do you feel like you have everything you need to do your volunteering work well?    1. How supported do you feel in your role?    2. Is there anything you feel that you would need that will help you in your volunteering role?    3. What do you think about the level of supervision in your volunteer work? 4. Why did you sign up as a volunteer?    1. Are those goals met?    2. What do you enjoy most about being a volunteer?    3. What do you enjoy least about being a volunteer?    4. How long do you see yourself continuing to volunteer?    5. In what way can the volunteering program be better improved to support you in your volunteering role? |
| **Program Objectives and Recommendations** | 1. What do you like most about this program? 2. Would you recommend this program to your friend? 3. Would you continue volunteering in this program? 4. How do you think the program can be improved? 5. Do you have any suggestions on what can be done to help people follow-up with their doctor? 6. Do you have any suggestions on what can be done to help people make healthier lifestyle choices? |
| **Additional Information** | 1. Is there anything else that we have not covered that you would like to share? |
