# Supplementary material for: Impact of Youth Community Health Volunteers on Community Health Screening Program Outcomes for Older Adults: Mixed Methods Evaluation Study
Source: J Med Internet Res. 2025 Dec 8;27:e75699. doi: 10.2196/75699 (PMC12685235; doi:10.2196/75699)
Supplement: Multimedia Appendix 4 [file jmir-v27-e75699-s004.docx]

**Table S4. Subgroup analysis of participants based on completion of the HealthStart Program**

|  | **Completed**  **(n = 158)** | **Dropout**  **(n = 34)** | **Test of difference or association** |
| --- | --- | --- | --- |
| Mean age, years (SD) | 67.7 (9.41) | 64.1 (10.1) | t(190) = 2.017, p = .045 |
| Gender (%)  Male  Female | 75 (47.5%)  83 (52.5%) | 17 (50%)  17 (50%) | $\chi^{2}\left( 1 \right)=0.072, p=.788$ |
| Race (%)  Chinese  Others | 140 (88.6%)  18 (11.4%) | 30 (88.2%)  4 (11.8%) | $\chi^{2}\left( 1 \right)=0.001, p=1.00$ |
| Marital Status (%)  Single  Married  Not reported | 112 (70.9%)  45 (28.5%)  1 (0.63%) | 21 (61.8%)  13 (38.2%) | $\chi^{2}\left( 1 \right)=1.211, p=.271$ |
| Highest Education Level (%)  No formal education  Primary education  Secondary Education  Tertiary Education | 17 (10.8%)  41 (25.9%)  65 (41.1%)  35 (22.2%) | 1 (2.94%)  11 (32.4%)  14 (41.2%)  8 (23.5%) | $\chi^{2}\left( 3 \right)=2.271, p=.518$ |
| Status of residential/Living Status (%)  Rent/Lodge  Own | 135 (85.4%)  23 (14.6%) | 30 (88.2%)  4 (11.8%) | $\chi^{2}\left( 1 \right)=0.023, p=.878$ |
| Residential Questionnaires (SD) Knowledge  PAM-13  eHEALs | 8.82 (1.60)  68.2 (17.5)  24.4 (10.7) | 8.29 (2.05)  75.2 (16.4)  26.7 (8.80) | t(190) = 1.635, p = .104  t(190) = -2.139, p = .034  t(190) = -1.177, p = .241 |
| *Note:* Independent sample t-test were used to test for differences in age, and residential questionnaires. Chi-square were used to test for association for the other variables. | | | |
