# Supplementary material for: Impact of Youth Community Health Volunteers on Community Health Screening Program Outcomes for Older Adults: Mixed Methods Evaluation Study
Source: J Med Internet Res. 2025 Dec 8;27:e75699. doi: 10.2196/75699 (PMC12685235; doi:10.2196/75699)
Supplement: Checklist 1 [file jmir-v27-e75699-s005.docx]

| **Guideline** | **Section and page number** |  |
| --- | --- | --- |
| Describe the justification for using a mixed methods approach to the research question | Methods – under Study Design and Rationale pages 4-5 |  |
| Describe the design in terms of the purpose, priority and sequence of methods | Methods – under Study Design and Rationale pages 4-5, under Data Integration page 6 and Figure 2: Flowchart of data collection and analyses |  |
| Describe each method in terms of sampling, data collection and analysis | Methods – under Quantitative Data Collection and Analysis pages 5-6 and Qualitative Data Collection and Analysis page 5 | |
| Describe any limitation of one method associated with the present of the other method | Strengths and Limitations pages 12-13 |  |
| Describe any insights gained from mixing or integrating methods | Table 3: Joint Display of Quantitative, Qualitative and Meta-Inference Findings, Discussion – under Primary Outcome page 11, Integrating Study Findings with the Theory of Change page 11-12 and Acceptability and Feasibility page 12 |  |
